# Collective knowledge and the dynamics of culture in chimpanzees

**DOI:** 10.1098/rstb.2020.0321

**Published:** 2022-01-31

**Authors:** Andrew Whiten, Rachel A. Harrison, Nicola McGuigan, Gillian L. Vale, Stuart K. Watson

**Affiliations:** ^1^ Centre for Social Learning and Cognitive Evolution, and Scottish Primate Research Group, School of Psychology and Neuroscience, University of St Andrews, South Street, St Andrews KY16 9JP, UK; ^2^ Department of Ecology and Evolution, University of Lausanne, Lausanne 1015, Switzerland; ^3^ School of Education and Social Sciences, University of the West of Scotland, Paisley PA1 2BE, UK; ^4^ Lester E Fisher Center for the Study and Conservation of Apes, Lincoln Park Zoo, Chicago, IL, USA; ^5^ Department of Evolutionary Biology and Environmental Studies, University of Zurich, Zurich, Switzerland; ^6^ Department of Comparative Language Science, University of Zurich, Zurich, Switzerland; ^7^ Center for the Interdisciplinary Study of Language Evolution, University of Zurich, Zurich, Switzerland

**Keywords:** collective knowledge, social learning, culture, cumulative culture, innovation, chimpanzee

## Abstract

Social learning in non-human primates has been studied experimentally for over 120 years, yet until the present century this was limited to what one individual learns from a single other. Evidence of group-wide traditions in the wild then highlighted the collective context for social learning, and broader ‘diffusion experiments’ have since demonstrated transmission at the community level. In the present article, we describe and set in comparative perspective three strands of our recent research that further explore the collective dimensions of culture and cumulative culture in chimpanzees. First, exposing small communities of chimpanzees to contexts incorporating increasingly challenging, but more rewarding tool use opportunities revealed solutions arising through the combination of different individuals' discoveries, spreading to become shared innovations. The second series of experiments yielded evidence of conformist changes from habitual techniques to alternatives displayed by a unanimous majority of others but implicating a form of quorum decision-making. Third, we found that between-group differences in social tolerance were associated with differential success in developing more complex tool use to exploit an increasingly inaccessible resource. We discuss the implications of this array of findings in the wider context of related studies of humans, other primates and non-primate species.

This article is part of a discussion meeting issue ‘The emergence of collective knowledge and cumulative culture in animals, humans and machines’.

## 1. Introduction

The study of animal culture has by now an approximately seven-decade history, encompassing a growing catalogue of vertebrate and invertebrate families, as well as a diversity of behavioural domains [1]. Alongside studies of the spread of milk-bottle opening by tits and regional birdsong dialects, primatology contributed importantly to the foundations of the field in the mid-twentieth century, through the now-famous studies of the spread of foraging innovations in provisioned Japanese macaque monkeys [1,2]. Later in the twentieth century, the study of wild primates, notably chimpanzees, began to play an influential role [3,4], and cultural primatology has since expanded to include scores of species and domains of behaviour [5].

Studies of that key building block of culture, social learning (learning from others) began much earlier, around the very start of the twentieth century [6] and continued all through it. But from today's perspective there was a remarkable disconnect between those efforts and the embryonic field of cultural primatology, for the scores of social learning experiments from 1901 [6] to 2003 [7,8] were (but for a single, oft-neglected exception [9] discussed below) restricted to one-to-one transmission only. Even within the complexities of human culture one-to-one transmission plays its part, but culture in the round entails much more. People learn from the collective knowledge suffusing their cultures, and varieties of many-to-one transmission exist alongside one-to-many and one-to-one transmission [10,11]. Moreover, cultural traditions are typically recognized only once innovations spread across populations, whether vertically between parents and offspring, horizontally within a generation, or obliquely between non-kin across generations [10]. When such innovations are transmitted repeatedly in one or more of these contexts with sufficient fidelity to maintain their identity as traditions [12], they instantiate a corpus of collective knowledge.

The limitations of the one-to-one transmission perspective have been increasingly recognized, in part spurred by fieldworkers' collation of evidence suggesting that monkeys and apes sustain long-lived cultures defined by group-wide traditions spanning multiple domains of behaviour [13–15]. By their nature, these field studies were limited in their ability to rigorously confirm an essential role for social learning in maintaining traditions, whereas experiments with appropriate control conditions can powerfully achieve this. Accordingly, a suite of experimental designs was engineered to examine the repeated transmission of behaviour patterns that defines culture. These ‘diffusion’ or ‘transmission’ experiments [16–18] were built particularly on two methodological foundations.

One foundation lay in an important advance in dyadic social learning experiments. For decades, the most basic form of these simply tested whether subjects who observed a model solve a task were, later, more likely to perform similar—although not necessarily identical—behaviours than subjects in a control condition that lacked a model and thus had to rely on individual-level learning only [7]. Later, ‘two-action’ designs improved on this approach by incorporating conditions in which each subject witnessed a model performing one of two (sometimes more) alternative acts on the same target objects [8,19]. Where subjects adopt the particular model variant they witness, one can then examine whether and to what extent these variants spread and are sustained as alternative traditions at the group level [16–18]. In chimpanzees, introducing such alternative models into whole groups provided the first experimental evidence in primates for the emergence of traditions in which the same tool is applied to the same foraging task, but using whichever of the different alternative techniques was seeded in the initial models [20].

The second influential foundation for these developments was a range of earlier diffusion experiments developed with non-primate species including rodents, birds and fishes, whose smaller size and large available sample sizes made them more tractable than great apes for such studies [16]. These studies pioneered experimental designs examining different aspects of diffusion, notably (i) ‘linear transmission chains’ of individuals, A–B–C, etc. that test for transmission across successive ‘cultural generations’; (ii) ‘open group diffusions’ that test for the spread of novel behaviour patterns by introducing one or more trained models into whole communities; and (iii) ‘replacement designs’ in which experienced individuals are removed from groups and naive individuals repeatedly introduced, testing the capacity of such collectives to sustain traditions through such simulated population turnovers [16]. Among primates, application of these methods has revealed differential transmission and spread of behavioural variants in chimpanzees, orangutans, colobus monkeys, capuchin monkeys and (wild) marmosets, as well as in a variety of birds, fishes and insects [16–18,21].

These experiments have been complemented by a growing corpus of new statistical techniques that identify social learning through the ways in which spontaneous innovations diffuse through objectively determined social networks [22]. These ‘network-based diffusion’ analyses have revealed cultural transmission in species like humpback whales, where diffusion experiments appear impossible [23]. Applications in primate research rely on relatively rare cases where novel behaviours emerge and their spread is documented, and to date this has been possible only for a modified form of sponge-tool invented by chimpanzees [24]. Close attention to further such opportunities will surely follow. These and other sophisticated statistical techniques are now being married with two-action and other transmission experimental designs outlined above, but conducted in the wild—a powerful combination that further addresses collective knowledge and culture [25,26].

In the present article, we survey a suite of our recent studies, principally with chimpanzees but also children, that explore linkages between collective knowledge and culture as a community phenomenon. First, we review a study in which evidence emerged of chimpanzees' tool-based solution to a foraging challenge being built through the integration of discoveries by different chimpanzees, the resultant innovation spreading to others to become an incipient tradition [27].

Second, we focus on conformity, which in broad terms occurs when a learner is swayed to adopt a behavioural option displayed by a majority of group-mates [28]. The phenomenon is thus inherently a collective one, insofar as a learner is influenced not by any single individual, but instead exploits the accumulated knowledge consolidated in ‘majority opinion’ expressed in their social world. Conformity can thus be regarded as a counterpart in social learning to collective decision-making in animal groups [29,30], such as when a swarm of bees opts for a particular new hive location once a majority are preferring to fly back and forth to it, sometimes described as ‘bee democracy’ [31]. Our recent studies suggest new twists in the way conformity operates among chimpanzees [32].

Third, we turn to the inter-related topics of social tolerance, supportive scaffolding and teaching. The scope for social learning in a community is modulated by the social dynamics operating between individuals, that may vary across a continuum from hostile (inhibiting cultural transmission) to highly tolerant (facilitating transmission) [33]. We describe our recent studies illustrating this variation [34].

## 2. Collective innovation, culture and cumulative culture

It has been commonly asserted that cumulative culture, in which successive cultural changes build on what went before [35–37], is the critical aspect that separates human culture from that of other species [38,39]. Based on over 30 years of detailed studies in the wild, Boesch [40] argued that to the contrary, multiple technologies of chimpanzees are consistent with a history of cumulative cultural evolution (CCE). If true, this suggests that although candidate instances in chimpanzees are minimal compared to what humans have achieved, human capacities for cumulative culture did not spring out of the blue, but evolved on foundations in our common ancestor that we can infer through studies of chimpanzees (it is, of course, a further question whether CCE occurs more widely across the animal kingdom). Boesch identifies CCE through comparing several complex technologies across multiple field sites where chimpanzees display habitual local differences in techniques, suggested to have been added on top of the repertoires they share more generally. Examples include: nut-cracking using natural hammer materials, arboreal and underground honey extraction, and subterranean termiting (see also [41,42]).

Such observations in the wild should be foundational to any consideration of cumulative culture in non-human species, but they suffer the limitation that a cumulative history is inferred rather than directly documented, as is possible in so many human examples, such as the evolution of axes, wheels and computers. Behavioural experiments that offer apes the potential for cumulation are, therefore, complementary in providing stepwise documentation of any such occurrences. In the first attempt to do so, wild-born juvenile chimpanzees in a Ugandan sanctuary were first shown and familiarized with application of a stick tool to simply dip honey from an artificial foraging device, then shown (by the same familiar, human model) that a more complex, multi-step application of the tool could make available all the honey and nuts inside [43]. Despite scores of opportunities, these youngsters stuck to their simple dipping method, whereas young human children later tested in an analogous way demonstrated cumulative learning of the more complex technique [44]. A later experiment obtained convergent results insofar as chimpanzees failed to copy a conspecific solving the third and most rewarding step in a series of progressively more challenging actions, instead tending to persevere with the simpler and less rewarding steps they had mastered, whereas young children often achieved all three steps [45].

These studies thus suggest that chimpanzees are more unlikely than humans to upgrade the complexity of their existing behaviours to match techniques shown by others to gain greater rewards. However, these are but two exploratory studies, that may have presented contexts not conducive to the capacities of interest, compared to those that characterize chimpanzees' natural environmental challenges. For example, in both cases the actions involved were small-scale and fiddly, accessible to childrens' fine-scale dexterity, but perhaps more challenging for less dexterous ape hands, lacking thumb opposability. In the wild, most candidates for CCE in chimpanzees involve larger scale actions that are relatively easy to see others perform. Experiments reviewed above demonstrating cultural transmission across groups have typically incorporated these characteristics.

Accordingly in a further experiment, we [27] offered groups of chimpanzees an array of potential and more readily manipulable tool materials, the novel application of which could relatively easily be witnessed by onlookers. In this study, each of six groups of chimpanzees was provided with a container of juice outside their enclosure mesh, and a variety of materials with which to gain the juice. These included a diversity of probes and straws that could be dipped in the juice, but the straws could also be used to suck up juice more efficiently. One object could do this most efficiently of all; a folded tube (long bendy tool (LBT)) that, to be used as a straw, had to be uncoiled to an appropriate configuration and the stop valve at one end unscrewed to remove it. Once the juice was depleted to a low level, this was the only tool that could deliver juice efficiently.

In three groups (Seeded) an experimenter showed a single chimpanzee how to use the LBT and allowed them to become expert in using it, while the other three groups (Unseeded) contained no such expert. We thus tested whether chimpanzees in the experimental, Seeded groups would, unlike in the earlier experimental CCE studies reviewed above [43,45], ‘step up’ from using the simpler dipping and straw-sucking approaches that were typically their initial responses, to acquire the more complex but also more efficient LBT technique displayed by the single existing expert in their group. The latter simulated the kind of ‘advanced’ innovator necessary for cumulative culture. The Unseeded groups acted as controls to test whether any LBT usage in the Seeded groups required observational learning or could instead be acquired by naive individuals.

In a first phase with 10 h of exposure, the efficiency of social learning was apparent [27]. Seven of the 18 chimpanzees (aside from models) in Seeded groups succeeded in creating a functional LBT and using it to suck juice, five others successfully used an LBT already made functional by another individual and all 18 attempted LBT usage. By contrast, just two of the 25 chimpanzees in Unseeded groups created a potentially functional LBT, and these two did not discover how to use it as a straw. A further 10 h of exposure including extensive video displays of models succeeding, had one further Seeded group chimpanzee but no individual from Unseeded groups creating and using an LBT.

**Figure 1.**
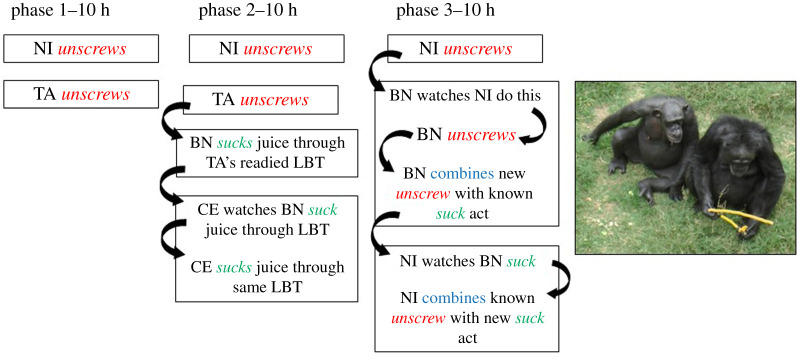
A case of collective knowledge generating more advanced behavioural solutions. In one of three groups not provided with a model demonstrating how to unfold a tube, unscrew a valve to open it and then shape it so it could be used as a straw to suck the juice from a container outside the enclosure mesh, this complex of actions nevertheless emerged through the collective actions of several individuals. In phase 1 two individuals unscrewed the valve but no more. In phase 2, collective solutions emerged through different individuals executing separate components of the whole sequence required. As chimpanzees further observed these in phase 3, two individuals combined them and thence mastered the task. For further explanation and discussion, see text.

In a third and final 10 h phase, we engineered a simulated ‘ecological stress’ event of the kind that may create a selection pressure for cumulative change: juice containers were fitted with lids permitting access only through small holes, so the only tools effective were now LBTs. At this stage, events particularly relevant to the topics of the current journal issue occurred in just one of the Unseeded groups (figure 1). In the earlier phases 1 and 2 an adult male NI and adult female TA had each unscrewed the valve of the LBT but failed to use it as a straw. However, in phase 2, a third chimpanzee, adult male BN, explored an LBT in which TA had already unscrewed the valve, and used it successfully as a straw. A fourth individual, CE, watched this and then did the same. Thus although no individual had performed the whole sequence necessary for LBT use, success was achieved through the collective activities of these four individuals. Then, in phase 3, BN watched NI unscrew a valve and repeat this, and he then combined it with the usage of the LBT as a straw that he had previously discovered using an LBT opened by TA. Through these collective events, BN had thus now mastered the entire sequence necessary for successful LBT use. NI then displayed the converse combinatorial progress, observing BN sucking juice and combining it with his existing knowledge of unscrewing the valve, hence also mastering the whole technique. We know from the Seeded condition that once LBT is shown by at least one individual, the technique will spread to constitute an incipient tradition.

Two additional control conditions allow us to build a more complete interpretation of what unfolded in these developments. In a ‘high level only’ condition, we provisioned a group of five chimpanzees with the circumstances of phase 3 from the start (the juice container was covered, requiring LBT use). This group thus lacked the opportunity over their 30 total hours of exposure to build their skills cumulatively, beginning with techniques such as sponging and probing with sticks and other materials, and sucking through simple straws. These subjects failed to achieve any successful LBT use; indeed, they did not manage to unscrew any valves. This contrasts with the successful achievements described above for individuals in one Unseeded group, (figure 1), who had become proficient in using a variety of simpler tools, including straws for sucking juice, through all of the prior 10 h phases.

**Figure 2.**
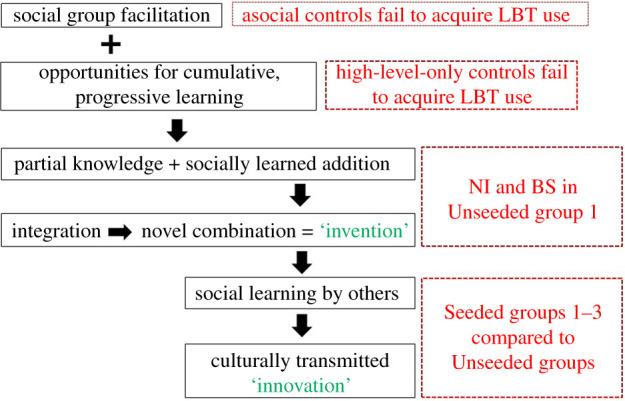
Schematic interpretation of findings in the ‘juice’ experiment [27]. The achievement of a culturally transmitted innovation builds through a series of precursor stages, indicated by core results shown in red. For explanation and discussion, see text.

A further ‘asocial’ control condition involved exposing five chimpanzees individually to phase 1 (1 h) and then phase 3 (1 h) conditions, thus providing them with sole, non-competitive access for a total of 10 h. One participant once unscrewed a single valve, but none achieved any successful LBT use. This suggests a facilitating effect of operating in a social group per se. Figure 2 integrates the results of these two control conditions with those for the main Seeded versus Unseeded conditions, and the events summarized in figure 1 for one of the Unseeded groups, to provide an overview of the conditions supporting the emergence of cumulative culture in chimpanzees.

Support for the basic facilitating effect of operating as a group, highlighted at the top of figure 2, comes from a separate, later study in which we presented chimpanzees (as well as, elsewhere, children) with opportunities to gain a range of reward levels in an environment offering opportunities for cumulative learning and culture [47]. Subjects faced a deliberately complex array of opportunities manifested in shelves differentiated by four levels, with the lowest level requiring the easiest actions to access but containing less preferred food rewards, and the highest level requiring the most challenging actions delivering the most desirable rewards. Level 1 required only manual actions to obtain the reward, level 2 required simple stick-tool-use, level 3 required a long tool to be made by combining or unfolding components, and level 4 required a hook to be added or unfolded at the end of the tool. In addition, at each level the reward initially placed in the middle of the shelf, could be guided using a finger (level 1) or tool (levels 2–4) to one of four different exit points. Each exit required a different approach to release the capsule, such as depressing a trap door or raising the capsule up to an opening. This complex array dubbed the ‘Small World’ thus offered 16 (4 × 4) different options for action. As in the juice experiment described above, chimpanzees were then tested in either small group or individual conditions.

We labelled as an ‘invention’ every first success in a group (or by the individual in a solo condition) concerning each of the 16 exit opportunities in the Small World. Interestingly, the eight groups tested achieved an average of 4.3 times as many inventions as the eight solo individuals, who achieved an average of only 1.5 inventions across 1 h of testing. Similarly, chimpanzees working in groups achieved as many as 11.5 more successful reward extractions in their first hour of testing as did the solo individuals. Thus, consistent with the results of the juice experiment described above, the collective work of these groups generated a diversity of task solutions not evident in the efforts of those acting individually, facilitating the availability of inventions as the ‘raw material’ to potentially build cumulative cultural advances.

In a parallel Small World experiment with young children, we found that those working in groups likewise generated more inventions and successes per unit of time than those working as lone individuals [48]. The greater availability of the ‘cultural raw material’ of inventions in groups of children was further associated with faster and more successful progress through higher levels in the Small World than in lone participants, with all groups succeeding at level 3 and only a handful of lone individuals reaching level 2. Additional evidence that such progress was facilitated by observational learning in the group led to the conclusion that a degree of cumulative cultural learning was operating in these groups of young children faced with the Small World.

By contrast, chimpanzees did not convert the enhanced availability of inventions in groups into cumulative cultural progress within the Small World. Groups did not generate more complex (higher level) solutions than individuals tested alone. Although three individuals in the groups managed to succeed by creating and using an elongated tool at level 3, only one other chimpanzee witnessing this achieved a similar task success; the discovery did not spread in the group, to become a shared innovation. This result is accordingly consistent with the findings of the earlier CCE experiments reviewed above [43,45] in which chimpanzees did not acquire a more complex and rewarding technique than they had already learned and habitually used, such as dipping for honey in the 2008 study [43]. However, this picture contrasts with the ‘juice’ study summarized in figures 1 and 2, in which collective contributions were converted into the mastery of a higher level combination of them ([27], compare with [49]).

Why might that be? We suggest that one reason may be that the Small World level 3 required tool construction, unlike the juice experiment, and the scope of chimpanzee tool manufacture as observed in the wild relies predominantly on reductive techniques like stripping leaves from stems, as opposed to constructive tool use. However, chimpanzees in the same colony have previously been shown to learn how to join sticks to create an elongated tool through observation [50]. An additional factor that may be in play is that unlike that study, chimpanzees in the Small World study could continue to gain rewards without making the tools necessary for level 3, by continuing to exploit rewards that were intermittently replenished at levels 1 and 2. Such a ‘satisficing’ explanation would be consistent with the context of the first CCE study, in which honey could always be gained by simple stick-dipping [43]. By contrast, the collective and cumulative effects we recorded in the juice experiment occurred only in the ‘ecological challenge’ context, when the lower level solutions were no longer available.

## 3. Social conformity

Conformity has long been studied as an influential factor in human social psychology [51] and in the foundational theories of human cultural evolution [52]. A recent review [28] distinguished three different manifestations of conformity, with the simplest—‘copy the majority’ being a preference to learn the most common among alternative behavioural options observed in others, either in the sense of the majority of individuals performing it or in that behaviour occurring at an overall higher frequency. More demanding forms, which tend to imply a more intense conformist tendency, are where (i) conformity is strong enough to overturn an existing, different (or even opposite) preference (‘Aschian conformity’, named after the social psychologist who performed the foundational studies [51]); or (ii) there is an exaggerated tendency to adopt the majority option, as when the probability of choosing an option is greater than its raw frequency in the observed population would predict (‘conformist bias’ [28,52]).

That the latter, conformity bias, might occur in animals, has historically been treated with some skepticism [53], but evidence for its operation has been adduced in recent experimental studies ranging from birds [46] to fruit flies [54]. Such studies tend to require substantial numbers of subjects to achieve the different frequencies required for testing, and we are not aware of such studies in apes or other primates.

The first cultural diffusion studies with primates suggested that Aschian conformity might be occurring because chimpanzees who initially adopted whichever of two tool-use techniques was experimentally seeded in their groups, but then went on to explore alternatives, later returned to preferring the option displayed by a majority of their group [20]. However, one alternative explanation, that a basic disposition to eventually focus on the first technique learned, could not be rejected [55]. What is ideally required is to present subjects at the outset with a choice between a majority behaviour versus a minority alternative. This was achieved in testing a basic ‘copy the majority’ bias by presenting children, chimpanzees and orangutans with a choice of gaining a reward by posting a token in one of three alternative boxes, after watching either three different individuals choose box A, or one individual choose box C three times. Both chimpanzees and children were found to choose box A, thus conforming to the majority, although orangutans did not [56].

The authors then tested whether subjects would reverse an earlier preference between target actions to conform to the majority preference they witnessed in others. Children demonstrated this stronger, Aschian form of conformity, but in this study [56] and two other experimental tests [57,58], chimpanzees did not.

However, these three studies each included conditions that may represent contexts militating against conformity [32]. In one study [57] the chimpanzees faced with an opposing majority were themselves in a small group sharing a minority behaviour, which in humans is known to reduce conformity compared to solo individuals' responses in the face of a unanimous majority [59]. In the other two studies, chimpanzees either had prior experience that the option they later saw preferred by a majority of others was unrewarded [56], or that it involved distasteful food [58]. To avoid these confounds, Watson *et al*. [32] first trained single chimpanzees to open a puzzle box using one technique, then introduced them into small groups in which the other individuals unanimously used a different technique, but not one the solo individual had earlier learned to avoid.

In this context, with a unanimous majority displaying a technique different to their own, the solo subjects did tend to conform, with four out of five chimpanzees displaying the majority option and most adopting it as their new preferred approach [32]. Only one in 23 majority individuals adopted the option initially shown by the minority individual. Similarly, neither control individuals tested by themselves nor individuals tested in pairs ever switched to the non-trained alternative, indicating a group effect rather than individual learning or copying the first option seen. Consistent with these experimental results, female chimpanzees dispersing to new communities in the wild have been reported to switch from their habitual forms of tool use to different approaches that are the norm in their new adopted group [60,61], a disposition also reported in dispersing male vervet monkeys [62] and great tits [46].

However, Watson *et al*. [32] reported an unexpected twist in how chimpanzees switched to majority options. These minority individuals did not wait to see what all or even the majority of their companions did but instead switched after observing the consistent approach displayed by just a subset of the group. The authors suggested that the underlying learning strategy might be akin to the finding that human subjects will, on the basis of similarly limited sampling, infer cultural norms and converge upon them [63]. Our findings may also reflect a phenomenon known as ‘quorum sensing’, which by analogy with the concept of a minimum quorum of attending committee members being sufficient to carry a vote, has been defined more generally as occurring when ‘threshold groups sizes trigger key changes in behaviour’ [64, p. 745]. To date, such effects appear to have been studied largely in fishes [65] and insects [66], but our results urge that more attention should be paid to it in primates and other taxa. Such a tendency may be particularly adaptive in species such as chimpanzees for whom, owing to their fission–fusion social structure, it may be impractical to sample the behaviour of every or even the majority of individuals in their community before making a decision; a unanimous majority of the currently available sample of them may suffice.

## 4. Social tolerance

Collective knowledge and collective memory require the existence of a collective. The great ape genera exhibit a very broad variety of community sizes and structures in which collective knowledge might emerge, from associations of just two to three individuals in some highly dispersed orangutan populations, to gorilla groups, to small fission–fusion parties of chimpanzees within a community spanning over a hundred individuals [67]. In addition, whatever the size and structure of the community, its members are often (excepting the mother–infant relationship) in competition for resources such as food or mating opportunities, diminishing tolerance of proximity. Finding that orangutans studied at Suaq Balimbing in Sumatra evidenced a more expansive cultural repertoire (especially in tool use) than other populations, and were also more likely to travel in small parties than alone, van Schaik [68] suggested that the degree of social tolerance typical of a species or social grouping (the probability for individuals to be in proximity to conspecifics around valuable resources with little or no aggression) may significantly shape the scope for cultural transmission.

The hypothesis was supported by an early finding that the extent of orangutans' specialist use of tools to extract insects from arboreal nests was well predicted by the size of small parties in which females and their offspring travelled [69]. Analysis of estimates of the extent of culturally transmitted forms of tool use in different communities of chimpanzees was likewise predicted by a composite measure of social tolerance, based on such variables as the percentage of individuals travelling alone as indicating lower tolerance [70].

Systematic and more comprehensive analyses of chimpanzee and orangutan cultural diversity across long-term study sites [13,14] later provided the opportunity to more rigorously test such relationships. For both species, the size of putative cultural repertoires of tool use and skills requiring significant practice for mastery was found to be significantly correlated with the mean percentage of time that individuals spent in association with one or more independent conspecifics (i.e. ignoring mother–offspring pairings) at distances less than 40–50 cm [71]. Of course, the underlying argument is not that tolerance is sufficient, but rather that its continuation after infancy is necessary.

These exploratory investigations were suggestive but limited. They rested on analyses of geographically widely distributed populations, making it difficult to reject a causal role for ecological or genetic factors [22]. We may also question whether they discriminate effects of the degree of social tolerance from the effects of opportunities for observational learning per se, such as those resulting from variations in party size. In a recent study [34], we were able to minimize such concerns by comparing two small communities of chimpanzees in adjacent enclosures in an African sanctuary, additionally applying newly developed and rigorous direct measures of social tolerance, independently of party size [72].

We focused on the role of individual and social learning in the face of cumulatively building challenges to obtain juice. Small groups of chimpanzees in two enclosures (group G3, 10 individuals; group G4, 12 individuals) were initially presented with a wide tube that contained juice, which could be obtained by dipping fingers or tool materials into the tube. To gauge behavioural flexibility in the face of progressive challenges—a key ingredient of cumulative culture—in a second phase the tube was narrowed, blocking manual access. The response of the two groups differed significantly. Analysis revealed that the odds of G4 using tools to obtain juice in an effective manner were over 31 times greater than for G3. Chimpanzees in G4 employed tools successfully in over 73% of their attempts, compared to only 27% in group G3.

Most interestingly, group G4 also developed a greater diversity of tool use in the narrow tube phase, employing 12 techniques, compared to just five in G3. Moreover in G4, as many as nine of these involved composite or combinatorial tool use, such as pushing a piece of absorbent cloth or sugarcane fibre into the tube using a stick, and then using the stick to fish this object out (a similar technique to obtain water was observed in wild chimpanzees at Bossou in Guinea, [73]). In G3 just two such techniques emerged.

We noted that a previous study developing systematic measures of social tolerance had reported greater tolerance in G4 compared to G3 [72]. Accordingly, we applied a complementary quantitative measure of tolerance to G3 and G4, based on behavioural interactions. This confirmed greater tolerance in G4. The most prominent ratios of positive indicators of tolerance in G4 compared to G3 were, in order, scrounging (14 times greater), co-action (touching the hand or tool of another individual already acting on the task) (3.9), tool transfers between individuals (3.3) and concurrent actions (simultaneous acts on the task) (2.6). Ratios of negative indicators of (in-) tolerance in G3 compared to G4 occurred in both displacement of others (1.8 times greater) and aggression (1.4). The correlations between what the two groups achieved in this context and their relative levels of social tolerance are clearly supportive of van Schaik's hypothesis that variations in social tolerance are likely to profoundly shape the scope for cultural transmission, as well as perhaps the emergence of a greater diversity of innovations (as described for G4). These may be important foundations for potential cumulative cultural change, not sufficient in themselves but operating in concert with other factors including those we focused on in the earlier sections above.

A recent study contrasting two wild chimpanzee populations, at Gombe in Tanzania and the Goualougo area in the Republic of the Congo, provides convergent findings [74]. Chimpanzees at both locations use tools to fish for termites, but at Gombe this involves using stems to probe above-ground mounds, whereas Goualougo chimpanzees employ significantly more complex techniques to harvest termites from subterranean nests. Individuals carrying long fishing stems in their mouths arrive at a known nest area and, instead of immediately using these, employ stout sticks to penetrate the ground almost vertically, creating a tunnel down to a nest. Sticks are sniffed to check when a nest has been penetrated. Once this is achieved, the end of the fishing stem is pulled through the teeth repeatedly to create a brush tip (which will stimulate more termites to bite it), then moulded to fit the tunnel. The stem is then carefully fed down the long tunnel and withdrawn with harvested termites attached.

The critical finding in relation to the topic under discussion here is that although youngsters at both locations beg for their mothers' fishing tools, mothers at Goualougo, where the more complex technologies are customary, are significantly more tolerant, with tool transfers from expert to novice as much as 5–8 times more frequent than at Gombe, probably supporting youngsters' mastery of the more complex technological culture that surrounds them [74].

## 5. Concluding discussion

We have described findings from three of our recent research projects that cast light on the core topics of this journal issue: collective knowledge, culture and cumulative culture. Interestingly, all of the three sets of findings we highlight were serendipitous discoveries, side-branches from the principal goals of their respective parent projects. The first, concerning collective innovation, was a chance occurrence in one of three control groups, rather than an experimental/control contrast the study was designed to focus on, but this perhaps underlines that the innovations that are key to both culture and cumulative culture may be rare occurrences that researchers are lucky to be able to document. Similarly the second study, concerning conformity, revealed an unexpected social dynamic akin to quorum decision-making. And the third, concerning the significance of social tolerance, was a finding incidental to the primary focus of the study, which was on chimpanzees' capacity for cumulative learning.

The findings of the first study demonstrated how a shared innovation may arise through two sequential manifestations of collective phenomena: first, the combination of different chimpanzees' exploratory actions, coupled with observational learning, to create a novel technology (LBT usage); and second, social learning by others from this, so collective knowledge of it becomes shared across a group [27]. We can summarize this in the conclusion that collective knowledge can be both an important cause and a consequence in the emergence of cumulative culture as in the human case [75]. As we noted above, this conclusion is based on a serendipitous set of observations, contrasting with many other reports of a lack of cumulative cultural change in chimpanzee social learning experiments [43,45,47]. This may imply that if instances of complex behaviour in the wild are indeed the results of cumulative culture that some propose [40–42], they may depend on processes of collective discovery and cultural transmission based on relatively rare inventive episodes that require long periods of time, multiple generations and/or large populations to generate them. They are thus inherently challenging to capture and document [24].

However, another timeframe over which similar phenomena may be in play is ontogeny. A recent review suggested three main phases of social learning occurring in most primates: first, a focus on the mother, second, on a progressively enlarging social network of other models, and third on new companions gained as adults disperse to join other groups [76]. In phase 2, for example, young male primates may apprentice themselves to adult males whose diet is different to their mothers' and hence acquire the collective knowledge spanning the two sexes [77]. In a quite different domain of competence, the scale of a juvenile chimpanzee's gestural repertoire has been shown to be enhanced in relation to the sociability of their mother, which opens up a greater collective gestural world to them [78].

The finding in our second research project was of conformity, which in the context of the present discussion we interpret as monitoring the predominant collective knowledge of one's companions, which are likely to represent optimal options to adopt because they are the result of multiple testing across the community [32]. It might be expected that this would militate against cultural change, cumulative or not, but what may be the only experimental study to directly address this reported to the contrary. Having studied the spread of experimentally seeded alternative foraging options (pushing a hatch to left or right) in large populations of great tits and implicating conformity in this [46], Aplin *et al*. [79] reversed the effective direction for the hatch. The authors reported that knowledge of the reversal spread over less than 14 days, through a combination of conformist and individual pay-off-sensitive individual reinforcement.

The third and final finding we highlighted was that greater tolerance in a small group of chimpanzees was associated with greater success in a challenging tool use task, including the generation of a more diverse set of potential technological solutions [34]. Thus as in the juice and LBT study we summarized, the collective aspects are twofold; tolerance may enhance the generation of the raw material (inventions) for potential cultural adoption or cumulative culture, and also the prospects for others adopting these through social learning. This is similarly illustrated by findings such as that greater gestural repertoires develop in the young of more sociable mothers [78]. Both collective invention and social transmission may be inhibited when tolerance is low, either as a secondary effect of high levels of resource competition or because individuals are motivated to conceal rather than share their special expertise—a possibility that perhaps begs more research attention. At the other end of the tolerance continuum, enhancement may occur when social learning is actively scaffolded, at the extreme amounting to an elementary investment in functional teaching [40,74].

## Data accessibility

This article has no additional data.

## References

[1] Whiten A. 2021 The burgeoning reach of animal culture. Science **372**, eabe6514. (10.1126/science.abe6514) 33795431

[2] Kawai M. 1965 New acquired pre-cultural behaviour of the natural troop of Japanese monkeys on Koshima Islet. Primates **2**, 1-30. (10.1007/BF01666109)

[3] Goodall JL. 1973 Cultural elements in a chimpanzee community. In Precultural primate behaviour (ed. EW. Menzel), pp. 144-184. Basel, Switzerland: Karger.

[4] McGrew WC. 1992 Chimpanzee material culture: implications for human evolution. Cambridge, UK: Cambridge University Press.

[5] Whiten A. 2012 Social learning, tradition and culture. In The evolution of primate societies (eds JC Mitani, J Call, PM Kappeler, RA Palombit, JB Silk), pp. 682-700. Chicago, IL: Chicago University Press.

[6] Thorndike EL. 1901 Mental life of monkeys. Psychol. Rev.: Monogr. Suppl. **III** 1-54.

[7] Tomasello M, Call J. 1997 Primate cognition. Oxford, UK: Oxford University Press.

[8] Whiten A, Horner V, Litchfield CA, Marshall-Pescini S. 2004 How do apes ape? Learn. Behav. **32**, 36-52. (10.3758/BF03196005) 15161139

[9] Menzel EW, Davonport RK, Rogers CM. 1972 Protocultural aspects of chimpanzees’ responsiveness to novel objects. Folia Primatol. **17**, 161-170. (10.1159/000155425) 5031297

[10] Cavalli-Sforza LL, Feldman MW. 1981 Cultural transmission and evolution: a quantitative approach. Princeton, NJ: Princeton University Press. 7300842

[11] Derex M, Beugin M-P, Godelle B, Raymond M. 2013 Experimental evidence for the influence of group size on cultural complexity. Nature **503**, 389-391. (10.1038/nature12774) 24226775

[12] Fragaszy DM, Perry S, (eds). 2003 The biology of traditions: models and evidence. Cambridge, UK: Cambridge University Press.

[13] Whiten A, Goodall J, McGrew WC, Nishida T, Reynolds V, Sugiyama Y, Tutin CEG, Wrangham RW, Boesch C. 1999 Cultures in chimpanzees. Nature **399**, 682-685. (10.1038/21415) 10385119

[14] van Schaik CP, Ancrenaz M, Borgen G, Galdikas B, Knott CD, Singleton I, Suzuki A, Utami SS, Merrill M. 2003 Orangutan cultures and the evolution of material culture. Science **299**, 102-105. (10.1126/science.1078004) 12511649

[15] Perry S et al. 2003 Social conventions in white-face capuchins monkeys: evidence for behavioral traditions in a neotropical primate. Curr. Anthropol. **44**, 241-268. (10.1086/345825)

[16] Whiten A, Mesoudi A. 2008 Establishing an experimental science of culture: animal social diffusion experiments. Phil. Trans. R. Soc. B **363**, 3477-3488. (10.1098/rstb.2008.0134) 18799418 PMC2607342

[17] Whiten A, Caldwell CA, Mesoudi A. 2016 Cultural diffusion in humans and other animals. Curr. Opin. Psychol. **8**, 15-21. (10.1016/j.copsyc.2015.09.002) 29506791

[18] Duboscq J, Romano V, MacIntosh A, Sueur C. 2016 Social information transmission in animals: lessons from studies of diffusion. Front. Psychol. **7**, 1147. (10.3389/fpsyg.2016.01147) 27540368 PMC4973104

[19] Whiten A, Custance DM, Gomez JC, Teixidor P, Bard KA. 1996 Imitative learning of artificial fruit processing in children (*Homo sapiens*) and chimpanzees (*Pan troglodytes*). J. Comp. Psychol. **110**, 3-14. (10.1037/0735-7036.110.1.3) 8851548

[20] Whiten A, Horner V, de Waal FBM. 2005 Conformity to cultural norms of tool use in chimpanzees. Nature **437**, 737-740. (10.1038/nature04047) 16113685

[21] Alem S, Perry CJ, Zhu X, Loukola OJ, Ingraham T, Søvik E, Chittka L. 2016 Associative mechanisms allow for social learning and cultural transmission of string pulling in an insect. PLoS Biol. **14**, e1002564. (10.1371/journal.pbio.1002564) 27701411 PMC5049772

[22] Hoppitt WJE, Laland KN. 2011 Detecting social learning using networks: a user's guide. Am. J. Primatol. **73**, 834-844. (10.1002/ajp.20920) 21246592

[23] Allen JA, Weinrich M, Hoppitt W, Rendell L. 2013 Network-based diffusion analysis reveals cultural transmission of lobtail feeding in humpback whales. Science **340**, 485-488. (10.1126/science.1231976) 23620054

[24] Hobaiter CP, Zuberbühler KT, Hoppit W, Gruber T. 2014 Social network analysis shows direct evidence for social learning of tool use in wild chimpanzees. PLoS Biol. **12**, e1001960. (10.1371/journal.pbio.1001960) 25268798 PMC4181963

[25] Barrett BJ, McElreath RL, Perry SE. 2017 Pay-off-based social learning underlies the diffusion of novel extractive foraging traditions in a wild primate. Proc. R. Soc. B **284**, 20170358. (10.1098/rspb.2017.0358) PMC5474070 28592681

[26] Canteloup C, Hoppitt W, van de Waal E. 2020 Wild primates copy higher-ranked individuals in a social transmission experiment. Nat. Commun. **11**, 459. (10.1038/s41467-019-14209-8) 31974385 PMC6978360

[27] Vale GL, Davis SJ, Lambeth SP, Schapiro SJ, Whiten A. 2017 Acquisition of a socially learned tool use sequence in chimpanzees: implications for cumulative culture. Evol. Hum. Behav. **38**, 635-644. (10.1016/j.evolhumbehav.2017.04.007) 29333058 PMC5765995

[28] Whiten A. 2019 Conformity and over-imitation: an integrative review of variant forms of hyper-reliance on social learning. Adv. Study Behav. **51**, 31-75. (10.1016/bs.asb.2018.12.003)

[29] Conradt L, Roper TJ. 2003 Group decision-making in animals. Nature **421**, 155-158. (10.1038/nature01294) 12520299

[30] Whiten A, Biro D, Bredeche N, Garland EC, Kirby S. 2021 The emergence of collective knowledge and cumulative culture in animals, humans and machines. Phil. Trans. R. Soc. B **377**, 20200306. (10.1098/rstb.2020.0306) PMC8666904 34894738

[31] Seeley TD. 2009 The wisdom of the hive. Cambridge, MA: Harvard University Press.

[32] Watson SK, Lambeth SP, Schapiro SJ, Whiten A. 2018 Chimpanzees prioritise social information over existing behaviours in a group context but not in dyads. Anim. Cogn. **21**, 407-418. (10.1007/s10071-018-1178-y) 29574554 PMC5908815

[33] van Schaik CP. 2003 Local traditions in orangutans and chimpanzees: social learning and social tolerance. In The biology of traditions (eds DM Fragaszy, S Perry), pp. 297-328. Cambridge, UK: Cambridge University Press.

[34] Harrison RA, van Leeuwen EJC, Whiten A. 2021 Chimpanzees’ behavioural flexibility, social tolerance and use of tool-composites in a progressively challenging foraging problem. iScience **24**, 102033. (10.1016/j.isci.2021.102033) 33521600 PMC7820130

[35] Mesoudi A, Thornton A. 2018 What is cumulative cultural evolution? Proc. R. Soc. B **285**, 20180712. (10.1098/rspb.2018.0712) PMC6015846 29899071

[36] Derex M. 2021 Human cumulative culture and the exploitation of natural phenomena. Phil. Trans. R. Soc. B **377**, 20200311. (10.1098/rstb.2020.0311) PMC8666902 34894732

[37] Gruber T, Chimento M, Aplin LM, Biro D. 2021 Efficiency fosters cumulative culture across species. Phil. Trans. R. Soc. B **377**, 20200308. (10.1098/rstb.2020.0308) PMC8666915 34894729

[38] Dean LG, Vale GL, Laland KN, Flynn E, Kendal RL. 2014 Human cumulative culture: a comparative perspective. Biol. Rev. **89**, 284-301. (10.1111/brv.12053) 24033987

[39] Henrich J. 2015 The secret of our success: how culture is driving human evolution, domesticating our species, and making us smarter. Princeton, NJ: Princeton University Press.

[40] Boesch C. 2012 Wild cultures: a comparison between chimpanzee and human cultures. Cambridge, UK: Cambridge University Press.

[41] Boesch C, Head J, Robbins M. 2009 Complex tool sets for honey extraction among chimpanzees in Loango National Park, Gabon. J. Hum. Evol. **56**, 560-569. (10.1016/j.jhevol.2009.04.001) 19457542

[42] Sanz C, Call J, Morgan D. 2009 Design complexity in termite-fishing tools of chimpanzees (*Pan troglodytes*). Biol. Lett. **5**, 293-296. (10.1098/rsbl.2008.0786) 19324641 PMC2679919

[43] Marshall-Pescini S, Whiten A. 2008 Chimpanzees (*Pan troglodytes*) and the question of cumulative culture: an experimental approach. Anim. Cogn. **11**, 449-456. (10.1007/s10071-007-0135-y) 18204869

[44] Whiten A, McGuigan H, Hopper LM, Marshall-Pescini S. 2009 Imitation, over-imitation, emulation and the scope of culture for child and chimpanzee. Phil. Trans. R. Soc. B **364**, 2417-2428. (10.1098/rstb.2009.0069) 19620112 PMC2865074

[45] Dean LG, Kendal RL, Schapiro SJ, Thierry B, Laland KN. 2012 Identification of the social and cognitive processes underlying human cumulative culture. Science **335**, 1114-1118. (10.1126/science.1213969) 22383851 PMC4676561

[46] Aplin LM, Farine DR, Morand-Ferron J, Cockburn A, Thornton A, Sheldon BC. 2015 Experimentally induced innovations lead to persistent culture via conformity in wild birds. Nature **518**, 538-541. (10.1038/nature13998) 25470065 PMC4344839

[47] Vale GL, McGuigan N, Burdett E, Lambeth SP, Lucas A, Rawlings B, Schapiro SJ, Watson SK, Whiten A. 2021 Why do chimpanzees have diverse behavioral repertoires but lack more complex cultures? Invention and social information use in a complex cumulative task. Evol. Hum. Behav. **42**, 247-258. (10.1016/j.evolhumbehav.2020.11.003)

[48] McGuigan N, Burdett E, Burgess V, Dean L, Lucas A, Vale G, Whiten A. 2017 Innovation and social transmission in experimental micro-societies: exploring the scope of cumulative culture in young children. Phil. Trans. R. Soc. B **372**, 20160425. (10.1098/rstb.2016.0425) 29061897 PMC5665812

[49] Wild S, Chimento M, McMahon K, Farine DR, Sheldon BC, Aplin LM. 2021 Complex foraging behaviours in wild birds emerge from social learning and recombination of components. Phil. Trans. R. Soc. B **377**, 20200307. (10.1098/rstb.2020.0307) PMC8666913 34894740

[50] Price EE, Lambeth SP, Schapiro SJ, Whiten A. 2009 A potent effect of observational learning on chimpanzee tool construction. Proc. R. Soc. B **276**, 3377-3383. (10.1098/rspb.2009.0640) PMC2817167 19570785

[51] Asch SE. 1956 Studies of independence and conformity. I. A minority of one against a unanimous majority. Psychol. Monogr. **70**, 1-70.

[52] Boyd R, Richerson P. 1985 Culture and the evolutionary process. Chicago, IL: University of Chicago Press.

[53] van Leeuwen EJ, Haun DB. 2013 Conformity in nonhuman primates: fad or fact? Evol. Hum. Behav. **34**, 1-7. (10.1016/j.evolhumbehav.2012.07.005)

[54] Danchin É et al. 2018 Cultural flies: conformist social learning in fruit flies predicts long-lasting mate-choice traditions. Science **362**, 1025-1030. (10.1126/science.aat1590) 30498121

[55] Haun DB, van Leeuwen EJ, Edelson MG. 2013 Majority influence in children and other animals. Dev. Cogn. Neurosci. **3**, 61-71. (10.1016/j.dcn.2012.09.003) 23245221 PMC6987688

[56] Haun DB, Rekers Y, Tomasello M. 2014 Great apes stick with what they know; children conform to others. Psychol. Sci. **25**, 2160-2167. (10.1177/0956797614553235) 25355648

[57] van Leeuwen EJ, Cronin KA, Schütte S, Call J, Haun DB. 2013 Chimpanzees (*Pan troglodytes*) flexibly adjust their behaviour in order to maximize payoffs, not to conform to majorities. PLoS ONE **8**, e80945. (10.1371/journal.pone.0080945) 24312252 PMC3842352

[58] Vale GL, Davis SJ, van de Waal E, Schapiro SJ, Lambeth SP, Whiten A. 2017 Lack of conformity to new local dietary preferences in migrating captive chimpanzees. Anim. Behav. **124**, 135-144. (10.1016/j.anbehav.2016.12.007) 29200465 PMC5705092

[59] Asch SE. 1951 Effects of group pressure upon the modification and distortion of judgments. In Groups, leadership, and men (ed. H Guetzkow), pp. 177-190. Pittsburgh, PA: Carnegie Press.

[60] Luncz LV, Boesch C. 2014 Tradition over trend: neighboring chimpanzee communities maintain differences in cultural behavior despite frequent immigration of adult females. Am. J. Primatol. **76**, 649-657. (10.1002/ajp.22259) 24482055

[61] Luncz L, Wittig RM, Boesch C. 2015 Primate archaeology reveals cultural transmission patterns in wild chimpanzees (*Pan troglodytes verus*). Phil. Trans. R. Soc. B **370**, 20140348. (10.1098/rstb.2014.0348) 26483527 PMC4614712

[62] van de Waal E, Borgeaud C, Whiten A. 2013 Potent social learning and conformity shape a wild primate's foraging decisions. Science **340**, 483-485. (10.1126/science.1232769) 23620053

[63] Rimal RN, Real K. 2005 How behaviors are influenced by perceived norms: a test of the theory of normative social behavior. Commun. Res. **32**, 389-414. (10.1177/0093650205275385)

[64] Sumpter DJ, Pratt SC. 2009 Quorum responses and consensus decision making. Phil. Trans. R. Soc. B **364**, 743-753. (10.1098/rstb.2008.0204) 19073480 PMC2689713

[65] Ward AJ, Krause J, Sumpter DJ. 2012 Quorum decision-making in foraging fish shoals. PLoS ONE **7**, e32411. (10.1371/journal.pone.0032411) 22412869 PMC3296701

[66] Pratt SC. 2005 Behavioral mechanisms of collective nest-site choice by the ant *Temnothorax curvispinosus*. Insectes Soc. **52**, 383-392. (10.1007/s00040-005-0823-z)

[67] Watts D. 2012 The apes: taxonomy, biogeography, life histories, and behavioral ecology. In The evolution of primate societies (eds JC Mitani, J Call, PM Kappeler, RA Palombit, JB Silk), pp. 113-142. Chicago, IL: Chicago University Press.

[68] van Schaik CP. 2003 Local traditions in orangutans and chimpanzees: social learning and social tolerance. In The biology of traditions: models and evidence (eds DM Fragaszy, S Perry), pp. 297-328. Cambridge, UK: Cambridge University Press.

[69] van Schaik CP, Fox EA, Fechtman LT. 2003 Individual variation in the rate of use of tree-hole tools among wild orangutans: implications for human evolution. J. Hum. Evol. **44**, 11-23. (10.1016/S0047-2484(02)00164-1) 12604301

[70] van Schaik CP, Deaner RO, Merrill MY. 1999 The conditions for tool use in primates: implications for the evolution of material culture. J. Hum. Evol. **36**, 719-741. (10.1006/jhev.1999.0304) 10330335

[71] Whiten A, van Schaik CP. 2007 The evolution of animal ‘cultures’ and social intelligence. Phil. Trans. R. Soc. B **362**, 603-620. (10.1098/rstb.2006.1998) 17255007 PMC2346520

[72] Cronin KA, Pieper BA, van Leeuwen EJ, Mundry R, Haun DB. 2014 Problem solving in the presence of others: how rank and relationship quality impact resource acquisition in chimpanzees (*Pan troglodytes*). PLoS ONE **9**, e93204. (10.1371/journal.pone.0093204) 24695486 PMC3973697

[73] Sugiyama Y. 1997 Social tradition and the use of tool-composites by wild chimpanzees. Evol. Anthropol. **6**, 23-27. (10.1002/(SICI)1520-6505(1997)6:1<23::AID-EVAN7>3.0.CO;2-X)

[74] Musgrave S, Lonsdorf E, Morgan D, Prestipino M, Bernstein-Kurtycz L, Mundry R, Sanz C. 2029 Teaching varies with task complexity in wild chimpanzees. Proc. Natl Acad. Sci. USA **117**, 969-976. (10.1073/pnas.1907476116) PMC6969499 31871167

[75] Migliano AB, Vinicius L. 2021 The origins of human cumulative culture: from the foraging niche to collective intelligence. Phil. Trans. R. Soc. B **377**, 20200317. (10.1098/rstb.2020.0317) PMC8666907 34894737

[76] Whiten A, van de Waal E. 2018 The pervasive role of social learning in primate lifetime development. Behav. Ecol. Sociobiol. **72**, UNSP 80. (10.1007/s00265-018-2489-3) PMC5934467 29755181

[77] Agostini I, Visalberghi E. 2005 Social influences on the acquisition of sex-typical foraging patterns by juveniles in a group of wild tufted capuchin monkeys (*Cebus nigritus*). Am. J. Primatol. **65**, 335-351. (10.1002/ajp.20120) 15834890

[78] Fröhlich M, Müller G, Zeiträg C, Wittig RM, Pika S. 2017 Gestural development of chimpanzees in the wild: the impact of interactional experience. Anim. Behav. **134**, 271-282. (10.1016/j.anbehav.2016.12.018)

[79] Aplin LM, Sheldon BC, McElreath R. 2017 Conformity does not perpetuate suboptimal traditions in a wild population of songbirds. Proc. Natl Acad. Sci. USA **114**, 7830-7845. (10.1073/pnas.1621067114) 28739943 PMC5544276

